# How much do pregnant women know about the importance of oral health in pregnancy? Questionnaire-based survey

**DOI:** 10.1186/s12884-023-05677-4

**Published:** 2023-05-13

**Authors:** Małgorzata Radwan-Oczko, Lidia Hirnle, Marta Szczepaniak, Irena Duś-Ilnicka

**Affiliations:** 1 Department of Oral Pathology, Wrocław Medical University, Ul. Krakowska 26, 50-425 Wrocław, Poland; 2 1st Department and Clinic of Gynecology and Obstetrics, Wrocław Medical University, Wrocław, Poland

**Keywords:** Pregnancy, Oral health awareness, Behavior, Parenting, Oral hygiene

## Abstract

**Background:**

Although pregnancy is a physiological process it causes hormonal changes that can also affect the oral cavity. Pregnancy increases the risk of gum disease inflammation and tooth caries which could affect the health of the developing baby. Proper oral health is crucial both for mother and her babies and is related with mothers’ awareness of this connection. The aim of this study was the self-assessment of women’s both oral health and oral health literacy as well as mothers’ awareness of the connection of oral health and pregnancy.

**Material and methods:**

In the study anonymous questionnaire was prepared and provided to be filled in by 200 mothers at the age from 19 to 44 y.o. who gave birth in the gynecological clinic. The questionnaire included demographic, and concerning the areas of oral health before and during pregnancy and after the childbirth questions.

**Results:**

Only 20% of the investigated women underwent the oral examination before the pregnancy and the next 38.5% underwent it intentionally when the pregnancy had been confirmed. As much as 24% of women pointed out lack of awareness of the importance of proper oral hygiene during pregnancy.

41.5% of investigated women declared complaints during the pregnancy concerning teeth or gums and 30.5% underwent dental treatment; 68%, brushed their teeth properly—twice a day; 32% of women observed deterioration of oral health state during the pregnancy. The knowledge of the importance of oral health during pregnancy presented by the majority of mothers was relatively proper, which was strongly connected with higher education status and living in big cities. A significant correlation between higher birth weight and more frequent daily tooth brushing was observed. Both higher frequency of problems concerning the oral cavity and dental treatment during pregnancy were significantly related to the younger age of mothers.

**Conclusions:**

The knowledge of women concerning of oral health on the management of pregnancy and development of fetus is still insufficient. Gynecologists should inquire pregnant women if they have done dental examination, and provide wider education about importance of oral health in pregnancy.

## Introduction

Although pregnancy is a physiological process, it causes hormonal changes that affect also the oral cavity. The presence and frequency of different oral problems of gums and teeth, mostly gingivitis, dental erosion, halitosis and pregnancy epulis have been described and are well known. In many clinical studies and meta-analyses the main association between the signs of periodontal disease and adverse pregnancy outcomes like preterm birth, low birth weight, preeclampsia, gestational diabetes [1], vulvovaginitis, premature rupture membranes has been presented [2–6].

The most frequent signs of gingival inflammation are related to increased levels of estrogen which disrupts proliferation and differentiation of cells and keratinization of epithelium, and increased levels of progesterone which changes vessels’ permeability and microcirculation in gingiva. Furthermore, in combination with oral pathological flora, an increased hormone level changes and decreases immune response is shown [7]. It leads to gums swelling and spontaneous or provoked gingival bleeding [8]. Although the plaque levels is declared to remain unchanged during the pregnancy, the gingival inflammation of pregnant women is significantly increased and peaked in the third trimester but dropped only at 3 months postpartum [7]. Finally untreated gingival inflammation, which can be reversible leads to periodontitis with periodontal attachment and bone loss and to the formation of periodontal pockets in the development periodontal diseases [9, 10]. Bacteremia, which indirectly triggers the hepatic acute phase response, enhances the production of cytokines, prostaglandins (PGE2), and interleukins (IL-6, IL-8) [11].

Special care of the oral cavity in women during pregnancy might be considered when food cravings to sweet food appear [12], influencing the change in the dental plaque formation pattern [13]. Proper healthy diet during pregnancy represents a positive influence on reducing the gingival and periodontal inflammation [12, 14]. As the sugar-rich diet has an influence on the bacterial load, its’ direct effect are dental caries, a common and costly disease in pregnant women [15]. Findings of the researchers from Pelotas show the far effect of dental caries in this group of patients, discussing that even depression is mediated by self-perception about oral health [16]. The authors show that the presence of depressive signals and symptoms was higher in pregnant women with dental caries experience, diverse severity of untreated dental caries, tooth loss, and filled tooth [16].

Different other factors have been discussed as important to the state of the oral cavity during the pregnancy and in the reproductive age. One of those is vitamin D levels in patients serum, that are considered to influence the composition of saliva, balance the caries activity, and stimulate the production of antimicrobial peptides, such as defensins and cathelicidin [17]. In reference to the reproductive age changes in the serum levels, the treatment with exogenous vitamin D have been related to better outcome of insulin, LDL-cholesterol and anti-Mullerian hormone levels in infertile women with polycystic ovary syndrome awaiting in vitro fertilization [18], and there are also reports suggesting that population approach aiming to eliminate the prevalence of vitamin D serum levels lower than 30 nmol/L in women of reproductive age, additionally facilitating reaching of the 50 nmol/L serum levels could be of a reasonable and safe goal [19]. In relation to this, vitamin D deficiency has been associated with the possible development of diverse complications among mothers [20] and pregnant women i.e. with the pregnancy related transient osteoporosis of the hip (PR-TOH) occurring in the third trimester [21].

Commonly appearing during the pregnancy granuloma gravidarum can be caused by an increased progesterone level in response to such irritants as bacteria, calculus, sharp elements of the broken teeth or food impaction. They are usually present in jaw in the first trimester, grow fast and retreat after childbirth. It could cause local bleeding while eating and toothbrushing [22]. It was also demonstrated, that pregnant women are at higher risk of erosions of enamel leading to hypersensitivity because of dissolving properties of gastric acid affecting the teeth during vomiting in the first trimester and acid reflux at the later stages [23]. Therefore, the maintenance of good oral health during the entire period of pregnancy is absolutely essential for general health of both mothers and their babies [5, 24, 25].

Many studies showed that healthier behaviors of future mothers depend on socioeconomic factors such as age, place of living, education level and number of children [26–28]. These factors, along with the self-assessment of women’s oral health and oral health literacy as well as awareness of the relationship between oral health state and pregnancy was the aim of this study.

## Materials and method

Study was performed by trained medical personnel who disseminated anonymous questionnaires. In this study an anonymous questionnaire-based survey was prepared and provided to be filled in paper version by the women who gave birth in the gynecological clinic. The questionnaire included 5 general demographic items and 11 questions concerning the oral health. The mothers provided answers without any help from the dentists, in order to collect real knowledge, without any suggestions, of women’s awareness of their oral health during the pregnancy. The study was approved by the Ethics Committee of Wrocław Medical University number Nr KB – 900/2012.

### Statistical analysis

For each continuous data mean (X), median (M), standard deviation (SD, range (min, max), lower and upper quartile (25Q, 75Q) were calculated. Statistical significance between means for different groups was calculated with the use of a one-way analysis of variance (ANOVA), alternatively using the non-parametric U Mann–Whitney test (for two groups) or Kruskal–Wallis test (for more than two groups), when the variances in groups were not homogeneous (the homogeneity of variance was determined by the Bartlett’s test).

Statistical significance between frequencies was calculated with the use of the chi-square test χ2df with Yate’s correction with corresponding degree of freedom df (df = (m-1)*(n-1), where m – number of rows, n – number of columns). A *p* value of less than 0.05 was required to reject the null hypothesis. Statistical analysis was performed using EPIINFO Ver. 7.2.3.1 software package.

## Results

Finally 200 questionnaires were collected from Caucasian women aged 31. 9 ± 5.3 on average. There were some questionnaires not fully completed, what could change the number of answers of some questions.

Only 170 mothers gave information about the length of pregnancy which was on average 38.9 ± 2.1 months. And only 172 mothers defined the baby’s birth weight, which was 3335.7 ± 508.2 g on average. The majority of women—61.5% (lack of 1.5% of answers) were from big cities. When education was considered, the majority of mothers had higher education—55.5%, and primary education had only 4.5% of respondents. Natural parturition was declared by 45% of mothers, 48.5% of them had caesarean section however 13 mothers did not answer this question. Nausea during pregnancy was indicated by 40 percent of women, as much as 58.5% did not have this condition and 1.5% of respondents did not answer this question. The data acquired from these general questions are presented in Table 1. Investigated oral related parameters are presented in Table 2.

**Table 1:** General questions—demographical and clinical parameters

*Women age*	*31.9 years, median 32.0 max 44.0 min 19*
*Week of delivery*	38.9 years, median 39.0, max 42.0 min 30,0
*Way of delivery*	
* • natural parturition*	45.0%
* • caesarean section*	48.5%
* • lack of answer*	6.5%
*Place of living*	
* • in the countryside*	13.5%
* • in small/medium town*	23.5%
* • in big town*	61.5%
* • lack of answer*	1.5%
*Educational status*	
* • primary and vocational education*	12%
* • secondary education*	32%
* • higher education*	55,5%
* • lack of answer*	0,5%

**Table 2:** Oral cavity related parameters performing

*Dental examination performing*	
*before pregnancy*	20%
*after confirmation of pregnancy*	38.5%
*Oral cavity state self-assessment before pregnancy:*	
* • very good*	30%
* • good*	51,5%
*Orthodontic treatment during pregnancy*	5%
*Nausea during pregnancy*	40%
*Lack of awareness of the importance of good oral hygiene during pregnancy*	24%
*Brushing teeth only once a day*	6%
*Oral problems during pregnancy*	
* • complains concerning teeth or gums*	41.5%
* • complains concerning dental hypersensitivity*	24.5%
* • gums bleeding*	37%
* • gingival overgrowth/ edema*	14.5%
* • dental treatment*	30.5%
* • deterioration of the oral cavity state*	32%

The first question related to the oral health in pregnancy was about the dental examination as important in pregnant women. When planning and preparing for the pregnancy only 20% of the investigated women underwent such examination and 38.5% of them had it done just after their pregnancy was confirmed. A statistically positive correlations between this examination and higher education of investigated women (chi-square test = 36.1 *p* ≤ 0.001) and living in the big city (chi-square test = 13.7 *p* ≤ 0.033) were observed. On the other hand women who lived in the countryside statistically less frequently underwent dental examination. As much as 41.5% of responders did not have the initial examination because 19.5% of women did not consider it’s necessity since they do not have any dental or oral problems and 22% did not have time or money for the oral cavity examination. When any problems or changes with their teeth or gums during the pregnancy were taken into consideration, the majority of women – 57% did not noticed them. There were lack of answer of 1.5% of whole investigated group. Women were asked for the self-assessment of the level of their oral health before pregnancy. In this investigated group 30% of them described it as very good, and 51.5% as good, and these states were indicated statistically more often by women with higher education. The next 17.5% of respondents felt discomfort with the calculus and the presence of small caries defects mainly in the group of women with primary and vocational education.(chi- square test = 14, *p* ≤ 0,024).

Furthermore, statistical differences concerning the assessment of the women’s oral health state and length of the pregnancy were observed. Longer time of pregnancy was correlated with worse self –assessment of oral health before pregnancy. During the time of pregnancy these feelings of oral cavity self-assessment changed and after the childbirth 20.5% of women described their oral health state as very good, and 47% as good, and these states were presented by women with higher education in 72.5% and 53.19% respectively. Moreover, 25% of the subjects described feelings of calculus and caries presence, 5.5% indicated their oral health status as bad and 4 mothers (2%) did not have or gave their opinion, mainly in the group of women with primary and vocational education, however, there was no strong statistical significance (chi-square test = 14.1, *p* ≤ 0.077).

**Fig. 1:**
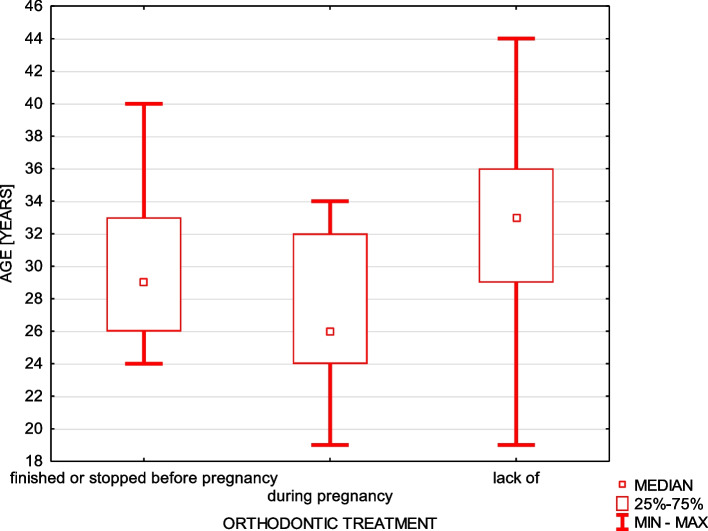
Orthodontic treatment during pregnancy in relation to the age

Only 5% of women underwent orthodontic treatment during entire or part of the pregnancy time and they were statistically younger (Fig. 1) and 12.5% removed orthodontic braces before the pregnancy.

Significantly more women who stopped their orthodontic treatment before pregnancy had higher education – 62.5% (chi-square test = 15.7 *p* ≤ 0.003), and there were no statistical differences in educational status concerning lack of orthodontic treatment. The presence of nausea during pregnancy was not statistically related to the use of orthodontic appliances (chi-square test = 1.97, *p* ≤ 0.374).

Regarding the information about the importance of good oral hygiene during pregnancy, only 16.5% of the investigated women knew about it before the pregnancy, 59.5% of respondents received this knowledge during their pregnancy and 24% of them were not aware of this knowledge until the end of pregnancy.

**Fig. 2:**
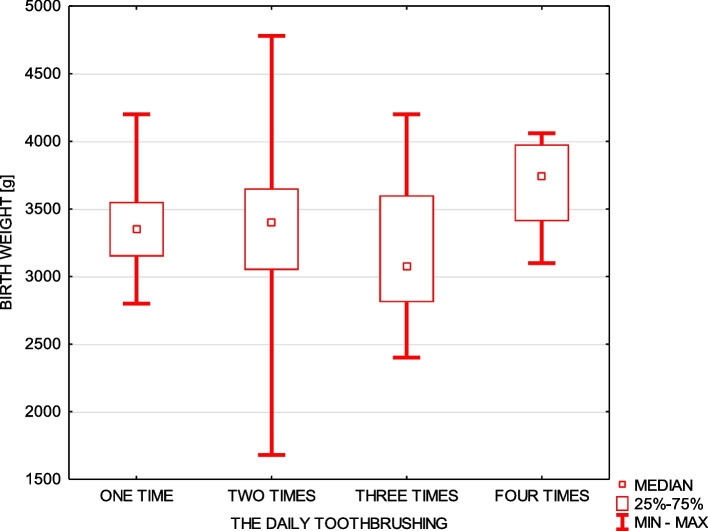
Relationship between the daily toothbrushing and child’s birth weight

**Fig. 3:**
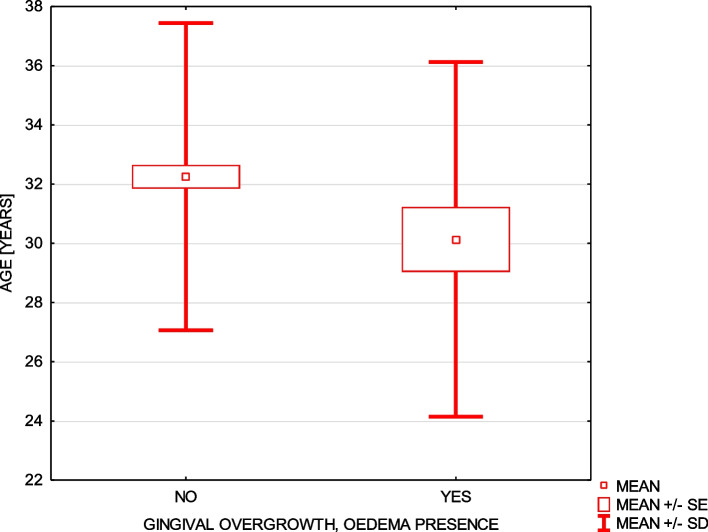
Relationship between the women’s age and gingival overgrowth

Furthermore, concerning the daily oral hygiene, 68% of respondents brushed their teeth twice a day, 21.5% three times daily, 6% brushed their teeth only once a day and 4.5% as much as four times a day. On the one hand the women with higher education status declared brushing teeth statistically more frequently – 87.5% of them. On the other hand women with primary and vocational education declared brushing their teeth mainly two times a day (70.83% of them) (chi-square test = 20,2 *p* ≤ 0,001). Statistically lower birth weight of newborns whose mothers declared brushing teeth only once a day and high birth weight of children whose mothers declared brushing teeth four times a day were observed (Fig. 2). Moreover, 37% of women indicated gum bleeding and this parameter was correlated with nausea during pregnancy (chi-square test = 10.5*p* ≤ 0.001). Gingival local overgrowth during pregnancy which was present in 14.5% of women, correlated with younger age (Fig. 3), and was significantly more often declared by women who had pregnancy nausea (chi-square test = 3.94 *p* ≤ 0.047) and by women who gave birth through the caesarean section ( chi-square test = 8.68 *p* ≤ 0.013).

**Fig. 4:**
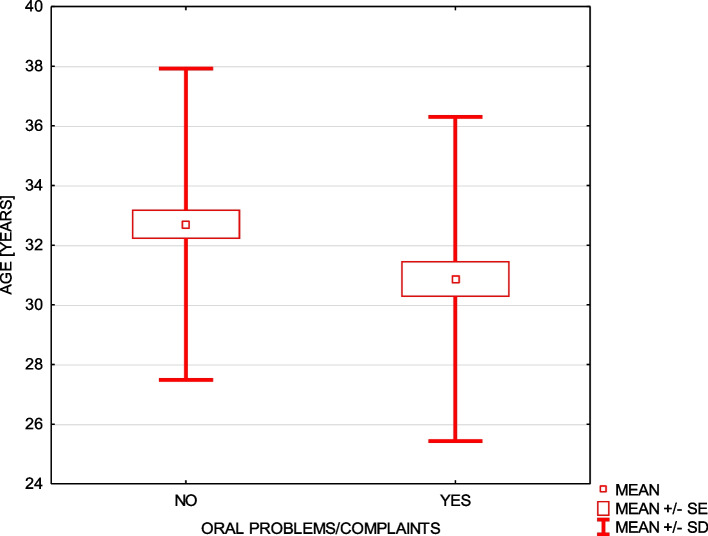
Frequency of the problems or complaints concerning the oral cavity during pregnancy in relation to the women’s age

Problems or complaints concerning teeth or gums were statistically more often described by younger women (Fig. 4) and by women experiencing nausea during pregnancy (chi-square test = 3.81 *p* ≤ 0.05). The signs of dental hypersensitivity confirmed 24.5% of women and 1% of all women did not answer this question.

**Fig. 5:**
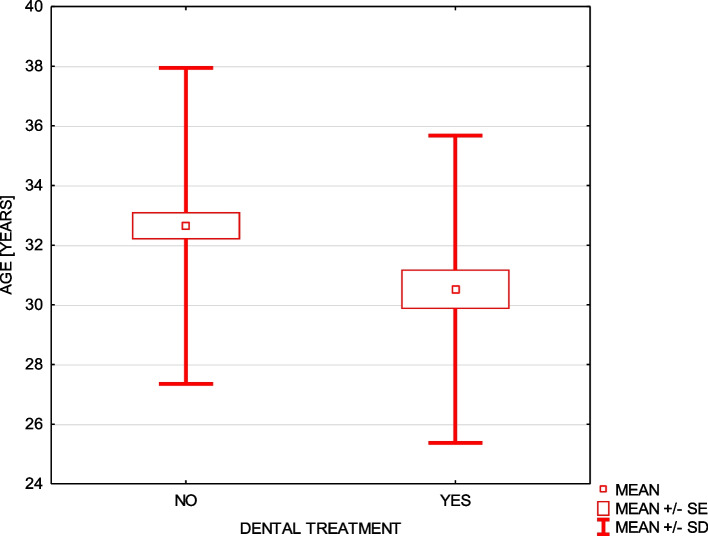
Dental treatment during the pregnancy in relation to the women’s age

As much as 30.5% of women had dental treatment during pregnancy and it was significantly more often performed in younger women (Fig. 5), however, 4 women (2%) did not answer this question. In the whole group of respondents, 5% had dental extraction, statistically more often in women living in the countryside (chi-square test = 6.30, *p* ≤ 0.043). Deterioration of the oral health state after pregnancy concluded 32% of mothers (Table 2).

## Discussion

Many assessments regarding the pregnant women oral health state and their knowledge of oral health in relation to the pregnancy have been performed in many populations from many countries. This topic seems to be very interesting and essential since the data showed the association between the oral care and oral health and both the general health, health of an unborn child and pregnancy outcomes [3–6]. Worldwide general health, dental, gynecological and obstetricians organizations or workgroups are involved in highlighting and discussing the importance of making pregnant women aware of the significance of their oral health [3, 24]. But it still seems this knowledge and awareness of both women and the knowledge providers are not sufficient, which has been presented in the published findings [22, 24, 26, 27].

Although this research presents and analyses only significant correlations between the investigated parameters, in general the obtained results are similar to other results described in this field. A lot of studies showed that healthier behaviors of future mothers depend on socioeconomic factors such as age, place of living, education level and number of children [26, 27, 29, 30]. The average age of the investigated group of women was 31.9 years and 45% of women gave a natural birth. The knowledge of the positive relationship between the appropriate oral health and correct course of pregnancy had only 16.5% of mothers before the pregnancy and as much as 59.5% got this information during the pregnancy, however, surprisingly, 24% of women still stated they did not have any awareness of these influences. In the work of Hom et al. [27], authors found a logical association between the oral health literacy and oral health knowledge. The level of health literacy influences seeking information about health, procedures and behaviors important for the maintenance of good health and this enhances health knowledge. In our study this phenomenon was also present. It should be pointed out that these 24% of women were not interested in oral health literacy, so their knowledge of oral health state related to complications was very low and underestimated.

Dental examination before or right after pregnancy confirmation was carried out in 58.5% of women, and they had higher educational status and lived in a big city. The study of Llena et al. [2] also confirmed such observations that better knowledge of oral health is related to the above determinants. As much as 81.5% of women described their oral cavity status before pregnancy as very good and good and this group of women had also higher education. Moreover, 24% of mothers reported lack of awareness of the importance of proper state of oral cavity during pregnancy and what is worth underlining, 19.5% of them considered this examination not necessary at all. Generally, health professionals must have awareness of the necessity of sharing the wide knowledge of the importance of oral health with pregnant women. Using the online questionnaires, Suri et.al. [31] compiled, with the help both of the dentist and obstetricians, the query evaluating the knowledge of the obstetricians about the association of periodontitis with preterm birth and birth weight. The authors noticed that more than 70% of respondents, who were quite young—the average age was 34.8 years, and 89% of whom were women, had proper knowledge of this issue. However, in the same group only 40% of respondents recommended dental examination and only 47% advised women to take care of oral health during pregnancy. Consequently, oral health literacy among pregnant women is still not sufficient, which was shown not only in this study. Even though the majority of obstetricians and gynecologists have proper and actual knowledge of the importance of oral health during pregnancy, they do not provide this information to the patients. What is more significant, they also do not require their patients to provide the confirmation of dental examination during pregnancy. Such examination, which constitutes a part of the assessment of general health in pregnancy, is not only recommended but also should be required at early pregnancy at the latest, or be an integral and obligatory part of pregnant care. The findings of Ghaffari et al. [22] were very important as they showed educational intervention to be effective and changing the awareness of pregnant women when it comes to their behavior concerning oral hygiene and oral health.

In this study the discomfort with the calculus or/and small caries defects during pregnancy were reported by 18.5% of women mainly with primary and vocational status education. General oral complaints concerning teeth or gums during pregnancy were reported by 41.5% women including gingival bleeding and the feeling of gingival overgrowth. It seems interesting that oral problems were significantly more often present in younger women and worse self-assessed oral health was more often related to the longer pregnancy time. The association between periodontitis and preterm birth is still not clear and the data are inconsistent. In our study we did not found any correlations between worse self-assessed oral health state and preterm birth. In the systemic review and current meta -analysis carried out by Manrigue- Corredor et al. with the participation of 10,215 women from America, Europe, Asia and Africa, the authors found the positive correlation between these parameters in 60% of 20 evaluated studies [32]. At the same time the authors underlined variability of the studies in the aspect of diagnosis of periodontitis and the presence of other risk factors as covariables.

As much as 32% of mothers stated deterioration of their state of oral cavity during the. pregnancy.

Nowadays, orthodontic treatment is very popular, especially among young women, sometimes also because of esthetic reasons [33, 34]. In our investigation 12.5% of women removed the orthodontic braces before the pregnancy as a result of termination of treatment or because of pregnancy. Only 5% of women who were significantly younger were under this treatment during the whole or part of the pregnancy period.

Brushing teeth twice a day is considered to be enough to maintain proper oral hygiene. In this survey, it was clearly visible that mothers with the higher educational status declared toothbrushing at least twice a day. On the one hand, 6% of mothers declared brushing their teeth only once a day, and there were significant correlations between the lower birth weight and such behavior. On the other hand, an association between a higher birth weight of newborn babies and toothbrushing four times a day was observed. Our results confirm other findings that show the influence of the daily toothbrushing on the oral hygiene and gingival inflammation which is associated with birth weight [35]. In the study of Gil [36], dental plaque level evaluated only supragingivally, was positively correlated with the periodontal parameters such as bleeding on probing, periodontal pockets depth and clinical attachment level. Moreover, the frequency of toothbrushing was negatively correlated with periodontal pockets depth and clinical attachment level. Furthermore, bleeding on probing and periodontal pockets depth were found as positively correlated with the CRP inflammatory marker, which confirms the fact that periodontal inflammation during pregnancy is the factor of a general importance.

As much as 37% of women complained about gingival bleeding and this parameter was positively correlated with nausea. Another complaint concerned the feeling of the gingival enlargement ( gingival edema or epulis) and it occurred in 14.5% of women who were significantly younger, more often had caesarean section and also felt nausea. It is well known that plaque-induced gingivitis is more often diagnosed in pregnant women because of the elevated levels of gestational hormones, which is transitional although influences gingival tissue response and changes immunological alteration. Therefore, gingival pockets, edema, or slight inflammatory overgrowth or pregnancy epulis are additionally the results of the hormonal–related gingival inflammation and not of the periodontal disease. An additional factor of the nausea can explain the presence of the described signs of gingival inflammation because of difficulties with the effective toothbrushing [24]. There are some discrepancies seen in the currently published meta-analyses assessing the relationship between the periodontal disease and adverse pregnancy outcomes [3, 4, 6]. In the work of Figuero et al. [3], pregnant women showed higher level of gingival inflammation when compared to the control group of non-pregnant women but without its correlation with salivary progesterone and estradiol levels. Authors also did not find any changes in IL-1β and PGE2 levels. These outcomes indicate no direct relationship between the gingivitis level and investigated parameters. However, other study showed that periodontal inflammation is not limited to the oral cavity and the periodic bacteremia and release of the endotoxins from the periodontopathogens can change the immune system response due to the production of proinflammatory cytokines particularly in women who show greater response to proinflammatory factors. Gil et al. [36] found a positive correlation between the CRP level and periodontal parameters such as pocket depth and bleeding on probing. The equivocal evidence concerning the positive influence of the periodontal treatment on adverse pregnancy outcomes was also described [5]. On the basis of meta-analysis of 11 trials, the authors [37] concluded that initial treatment of periodontal disease cannot be considered as an efficient way of decreasing the incidence of preterm birth. The cited authors underline that this treatment is not most important in the protection of adverse pregnancy outcomes.

Surprisingly, 30.5% of women who underwent dental treatment during the pregnancy were significantly younger. There was no exact information in the questionnaire what kind of treatment was conducted. Furthermore, five women who had tooth extraction were significantly more often from the countryside population. Some dentist may by unwilling not only to treat pregnant women, but also to carry out the oral examination because of liability concerns. On the other hand, the liability resulting from the lack of treatment or consultation of pregnant patients may be higher and unpredictable. Physicians, obstetricians and dentists should always spread the information about the necessity and safety of dental examination, particularly among the young women. A proper approach to clear communication and education related to the proper oral health and its connection with general health of a pregnant women and fetuses are of great importance. Additionally, very simple, but important advices concern the proper toothbrushing, using mouth rinsing, flossing and other recommendations which are dedicated at individual stages. Only the described attitude with collaborative relationship between the medical doctor and pregnant woman seem to improve both the oral health literacy and oral health knowledge, which is of the utmost importance to everybody.

We shall notice a possible limitation of this study, because of only self-reporting assessment of the oral cavity parameters. Even though this method is generally accepted, the clinical assessment of the above parameters would be more explicit.

It is worth underlining that the self-assessment avoided bias, which could occur during the nice direct contact with the patients, and collected information gathered data about oral health state from three periods—before, during and after the pregnancy.

## Conclusions

**Fig. 6:**
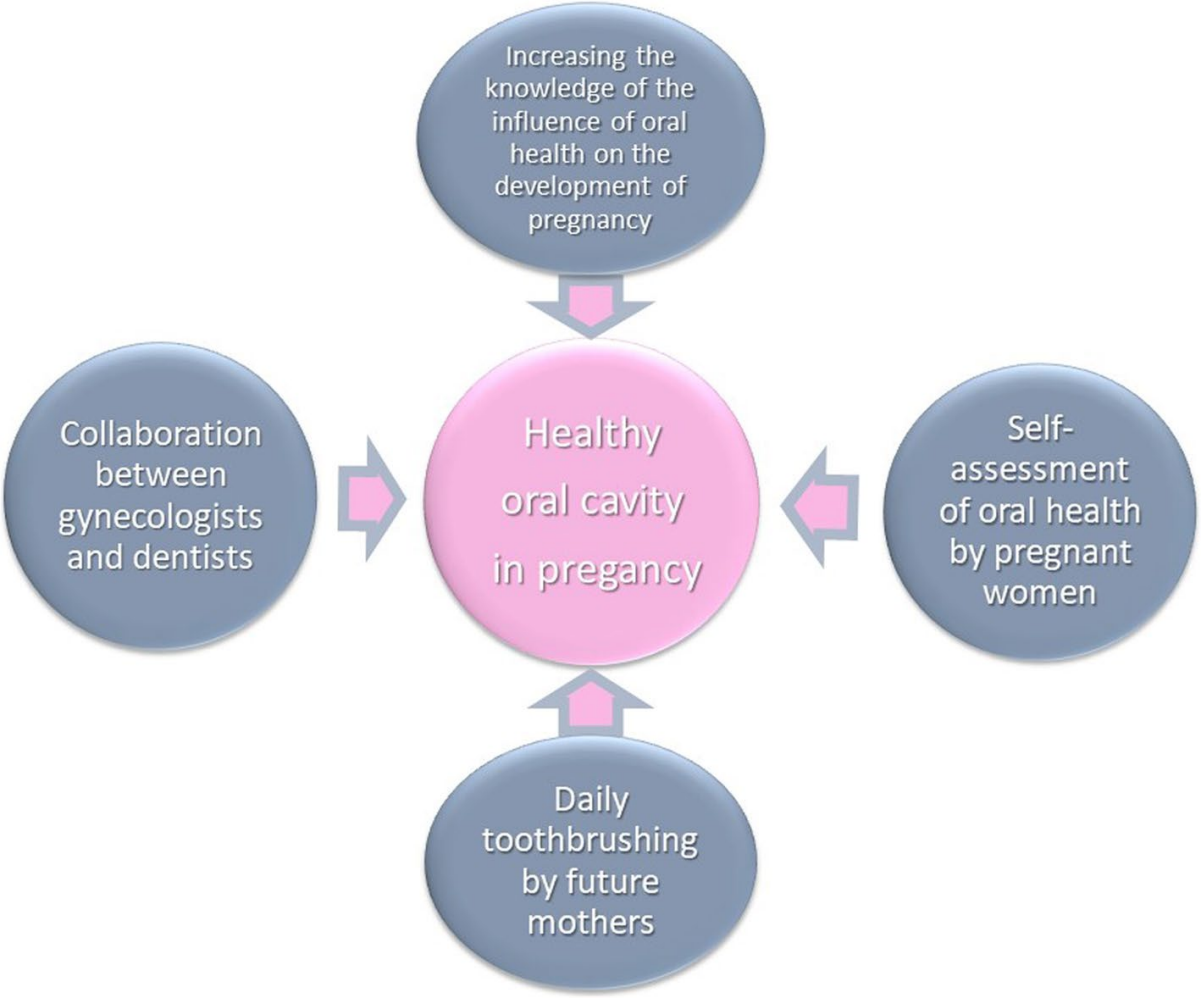
Proper treatment of the oral health status and collaboration between medical specialist, might provide the improvement of oral health in pregnant women

The relation between the longer duration of pregnancy and self- assessed worse oral health before pregnancy has been shown. This particularly concerned women with lower educational status. A correlation between daily toothbrushing and birth weight of newborns was found. Health-related behaviors and life-style of future mothers depend on socio-economic factors. Doctors should identify groups of women at increased risk (women with lower economic status, living in the countryside) and provide better education and medical care [38]. The knowledge of women about the impact of oral health on the development of pregnancy and the fetus is still insufficient. In addition to educational activities that aim at increasing women's knowledge of the impact of the oral health state on the development of pregnancy, gynecologists should inquire whether pregnant women have done the appropriate examination. The self-assessment of oral health by pregnant women may be the first step in accelerating their health-promoting activities, Fig. 6.

## Data Availability

The datasets supporting the conclusions of this article are included within the article.
